# Consecutive Daily Measurements of Luminal Concentrations of Lactate in the Rectum in Septic Shock Patients

**DOI:** 10.1155/2012/504096

**Published:** 2012-02-22

**Authors:** Michael Ibsen, Jørgen Wiis, Tina Waldau, Anders Perner

**Affiliations:** ^1^Intensive CareUnit 4131, University of Copenhagen, Rigshospitalet, Blegdamsvej 9, 2100 Copenhagen, Denmark; ^2^Department of Anaesthesia and Intensive Care, Herlev Hospital, University of Copenhagen, 2730 Herlev, Denmark

## Abstract

In a recent study we found no difference in the concentrations of luminal lactate in the rectum between nonsurvivors and survivors in early septic shock (<24 h). This study was initiated to investigate if there are any changes in the concentrations of luminal lactate in the rectum during the first 3 days of septic shock and possible differences between nonsurvivors and survivors. *Methods*. We studied 22 patients with septic shock in this observational study. Six to 24 h after the onset of septic shock the concentration of lactate in the rectal lumen was estimated by 4 h equilibrium dialysis (day 1). The rectal dialysis was repeated on day 2 and day 3. *Results*. The concentration of lactate in the rectal lumen did not change over the 3 days in neither nonsurvivors nor survivors. Rectal luminal and arterial lactate concentrations were not different. *Conclusion*. There was no change in the concentration of lactate in the rectal lumen over time in patients with septic shock. Also, there was no difference between nonsurvivors and survivors.

## 1. Introduction

Resuscitation of patients with septic shock is most often guided by only global parameters such as mean arterial pressure (MAP), central venous pressure (CVP), central venous oxygen saturation (ScvO_2_), and arterial lactate [1, 2]. However, patients who appears initially to be adequately resuscitated as judged by global parameters may later develop multiple organ failure with fatal outcome [3]. Inability of splanchnic blood flow to meet metabolic demands has been proposed to be one factor in the development and persistence of multiple organ failure in such patients [3, 4]. Therefore, different techniques have been used to assess splanchnic blood flow and metabolism [5–8].

 Equilibrium dialysis is a simple, minimally invasive method for the estimation of the concentration of lactate luminally in the rectum and the method was first used to show differences in electrolyte transport and production of inflammatory markers in patients with inflammatory bowel disease [9–11]. Using this method in patients with severe sepsis and septic shock persisting for more than 24 h we have previously shown that luminal concentrations of lactate in the rectum correlate with large bowel permeability [12] and disease severity and outcome [13] indicating pathophysiological relevance. However, in a larger study of patients with septic shock for less than 24 h we observed low luminal rectal concentrations and no relation between concentrations of lactate in the rectal lumen and mortality [14]. Taken together, we have found higher concentrations of lactate in the rectal lumen in patients with septic shock for more than 24 h than in those patients with septic shock for less than 24 h. These observations suggested that the rectal lactate concentration could change over time in some patients and potentially be a marker of outcome.

 Therefore, the aim of the present study was to perform daily rectal equilibrium dialysis for the first three days in patients with septic shock to investigate if the concentration of lactate in the rectal lumen changed during this period of time.

## 2. Materials and Methods

 The 22 patients were enrolled at the general intensive care units of Rigshospitalet and Herlev Hospital, University of Copenhagen, Denmark and the population included both surgical and medical patients. The regional ethics committee approved the study protocol and informed written consent was obtained from the patient or the next of kin. The investigation was registered at http://www.clinicaltrials.gov/ (no. NCT00197938).

### 2.1. Design

 This was a prospective, observational, pilot study with daily consecutive measurements of luminal rectal lactate concentrations in patients on the first 3 days of septic shock.

 Patients were enrolled consecutively when meeting the following inclusion criteria: (a) septic shock according to consensus criteria [15] and (b) infusion of vasopressors for 6–24 h. By not including patients in the first 6 h of vasopressor treatment the aim was to exclude patients needing only a short (<6 h) period of vasopressor support during initial resuscitation. All patients were resuscitated according to the principles of the Surviving Sepsis Campaign Guidelines and early goal-directed therapy. Thus, the first rectal equilibrium dialysis was performed 6–24 h after onset of shock (day 1) and repeated 24 h (day 2) and 48 h (day 3) after the initial dialysis.

 Exclusion criteria were age less than 18 years, vasopressor treatment for more than 24 h, rectal bleeding or pathology, cardiac arrest during the current episode of sepsis, previous episode of septic shock within current ICU admission or limitations or impending withdrawal of active therapy of the patient. Patients were treated according to local guidelines and clinicians were unaware of the results of the rectal dialysis.

### 2.2. Data Registration

 The following data were registered in all patients: arterial lactate, mean arterial blood pressure (MAP), central venous oxygen saturation (ScvO_2_), noradrenaline dose, and intra-abdominal pressure (IAP). To calculate the group medians of parameters other than concentrations of rectal lactate the mean of the registrations done before and after the 4 h of rectal dialysis in individual patients were used. Simplified acute physiology scores (SAPS) II were calculated based on values of the first 24 h after ICU admission and sequential organ failure assessment (SOFA) scores were calculated at inclusion and daily thereafter until either death or discharge from the ICU. Thirty-day mortality was obtained from hospital registries.

### 2.3. Rectal Equilibrium Dialysis

 Measurement of rectal luminal lactate was done as previously described [16, 17]. In brief, a 12 cm long bag of dialysis tubing (semipermeable cellulose, molecular weight cut-off 12 kDa, Sigma, St. Louis, MO, USA) was filled with 4 mL of 6% dextran 70 in saline (Macrodex, MEDA Group, Solna, Sweden) and closed over 5 cm of Tygon tube (Cole-Parmer Instruments Company, Vernon Hills, IL, USA) with a three-way stopcock at the distal end to allow sampling. Once filled, the bag is firm and can be easily inserted into the rectal lumen after digital exploration. Part of the Tygon tube and the three-way stopcock will then protrude from the anus. The dialysate was sampled after 4 h of dialysis, since 90–95% equilibrium with the lactate concentration in the surrounding medium is obtained at this time point [17]. Dialysates were analysed immediately at the study sites using standard blood gas autoanalysers (ABL 725, Radiometer, Copenhagen, Denmark), which were calibrated according to the manufacturer's instructions. This analyser is stereospecific and measures only concentrations of L-lactate.

### 2.4. Statistics

 Continuous variables are presented as medians (25th–75th percentiles) unless stated otherwise. The Mann-Whitney test or Fisher's exact test were performed where appropriate. The Friedman test (repeated measures) was used when analysing three paired groups (e.g., changes in concentrations of luminal rectal lactate over 3 days) and Wilcoxon's signed rank test was used when analysing two paired groups of observations (e.g., changes in concentrations of luminal rectal lactate over 2 days in those patients with only 2 dialysis periods). All analyses were done using GraphPad Prism v. 4.00 (GraphPad Software, San Diego, CA, USA). Values of *P* < 0.05 (two-tailed) were considered significant.

## 3. Results

 The overall 30-day mortality of the study population was 23% (5 nonsurvivors and 17 survivors). The characteristics of the 22 patients are shown in Table 1. All patients underwent rectal equilibrium dialysis in 2 consecutive days, but only 15 patients underwent rectal equilibrium dialysis in all 3 days (3 patients were discharged or transferred to another ICU, 1 patient died, and 3 patients had profuse diarrhoea on the 3rd day, so that the dialysis bag slipped out of the rectum).

### 3.1. Nonsurvivors and Survivors

 There were no differences in the concentrations of lactate in the rectal lumen between nonsurvivors and survivors on any day. On day 1 the concentration of rectal lactate was 2.4 (1.3–7.5) mmol/L in nonsurvivors and 2.1 (1.2–4.4) mmol/L in survivors (*P* = 0.58) see Table 2. On day 2 the rectal concentrations of lactate were 3.2 (1.7–4.2) mmol/L and 2.1 (1.1–3.4) mmol/L (*P* = 0.39) and on day 3 the concentrations were 2.9 (1.5–3.0) mmol/L and 2.3 (0.9–3.0) mmol/L (*P* = 0.61), respectively, (Table 2).

**Figure 1:**
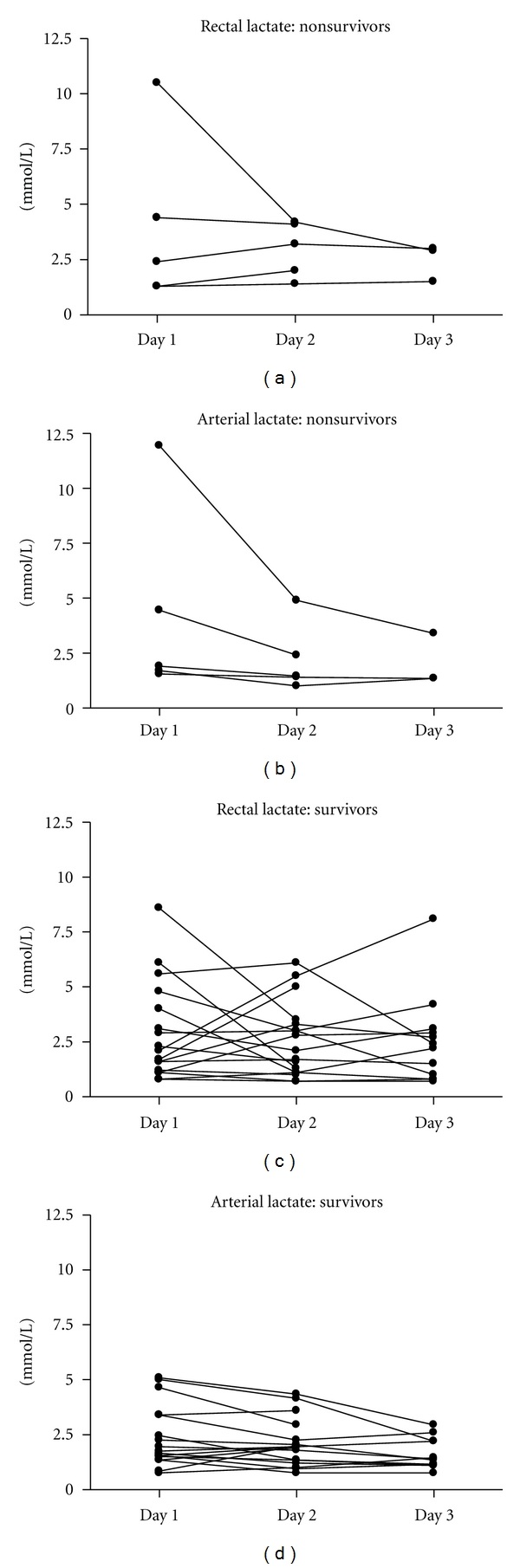
Rectal luminal and arterial concentrations of lactate in nonsurvivors and survivors of septic shock. There was no significant difference between the groups on any day (Mann-Whitney test) or within the groups over the days (Wilcoxon's signed rank test or Friedman test comparing paired values within groups over 2 or 3 days, resp.). See also Table 2 and text.

 Neither were there any changes in the actual concentrations of lactate in the rectal lumen over the 3 days in neither nonsurvivors nor survivors; see Figure 1.

 Similarly, there were no difference in arterial values of lactate between the groups of nonsurvivors or survivors or within the groups over the days; see Table 2 and Figure 1.

### 3.2. Changes in Luminal Rectal Lactate over Time

 Data were stratified according to patients with an increase or a decrease/no change in the concentration of lactate in the rectal lumen from day 1 to day 2 and/or from day 2 to day 3; see Table 3. Eleven patients had an increase in the concentration of luminal rectal lactate from day 1 to day 2 with a median increase of 0.7 (0.1–1.7) mmol/L and 7 patients had an increase from day 2 to day 3 (1.0 (0.1–1.2) mmol/L). Eleven patients had a decrease in the concentration of rectal lactate from day 1 to day 2 (−1.0 (−0.3–−4.8) mmol/L) and 8 patients a decrease from day 2 to day 3 (−0.5 (−0.2–−1.7) mmol/L). There were no difference in SAPS II, SOFA score at inclusion or day 5 between the groups with an increase or a decrease/no change in rectal lactate either from day 1 to day 2 or from day 2 to day 3.

### 3.3. Rectal versus Arterial Concentration of Lactate

**Figure 2:**
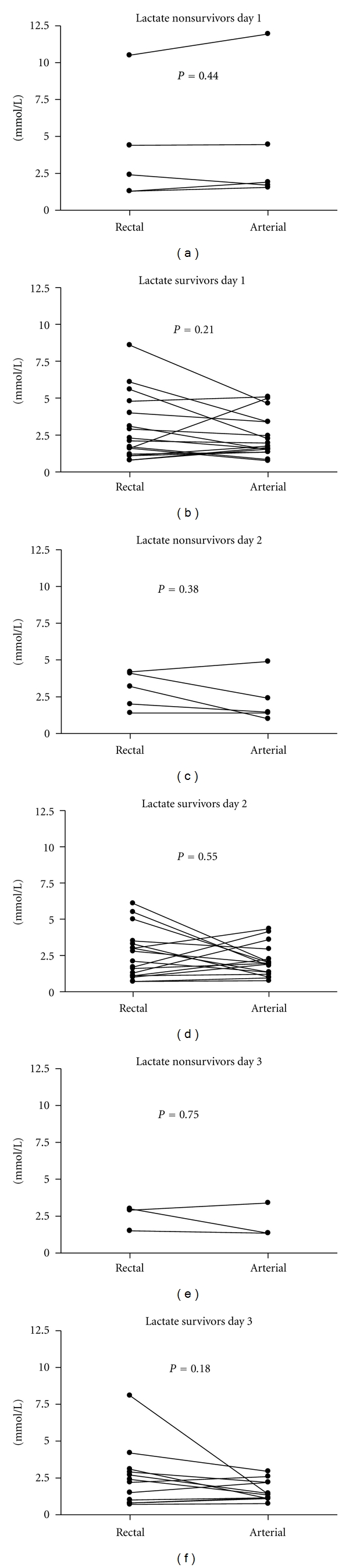
Rectal and arterial concentrations of lactate in nonsurvivors and survivors of septic shock. There were no differences using Wilcoxon's signed rank test.

 The luminal rectal and arterial concentrations of lactate did not differ significantly on any day in any group, see Figure 2.

The rectal-arterial gradient (delta-lactate) was not different in nonsurvivors on any day and did not change over the days in either group; see Table 2. There was no correlation between MAP, noradrenaline dose, intraabdominal pressure or ScvO_2_, and rectal lactate concentrations in any group at any time.

Ninety-day mortality was 41% (9 nonsurvivors and 13 survivors). Results were unchanged when data were analysed according to 90-day mortality (data not shown).

## 4. Discussion

 There were four main findings of this study. Firstly, the concentrations of lactate in the rectal lumen did not change over the first 3 days in patients with septic shock. Secondly, there was no difference in the rectal lactate concentrations between nonsurvivors and survivors. Thirdly, there were no differences in SAPS II and SOFA scores at inclusion or day 5 in those patients with increasing concentrations of lactate in the rectal lumen compared with those patients with a decrease/no change and fourthly, there was no significant difference between luminal rectal and arterial concentrations of lactate.

 These findings raise some important questions. Why this discrepancy of the observations of these later studies and the previous study regarding an association between concentrations of lactate in the rectal lumen and outcome? Mortality is a “hard” outcome parameter often requiring larger populations to establish a significant difference, so it is perhaps not surprising that we did not observe any difference between nonsurvivors and survivors in the present small population. However, in the study of early septic shock we investigated 130 patients [14] and significant differences in mortality have been seen before in patient populations of this smaller size, both by ourselves and others [13, 18].

 In the previous cohort [13], in which the mortality was 48% (11 of 23 patients), we observed significantly higher concentrations of lactate in the rectal lumen in nonsurvivors compared to survivors of severe sepsis or septic shock. In that study the lactate concentrations in the rectal lumen were also higher than arterial concentrations and the difference were more pronounced (5.0 (0.9–11.8) versus 2.2 (0.4–4.9) mmol/L; *P* < 0.0001) than the difference in the arterial concentrations (3.8 (1.7–7.0) versus 1.6 (0.5–3.6) mmol/L; *P* < 0.01) between nonsurvivors and survivors. These findings suggested that the concentration of lactate in the rectal lumen could be an important marker of regional metabolic dysfunction of the gut and distinctly different than arterial concentrations of lactate. Therefore that study was followed by the study of patients with early (6–24 h) septic shock [14], which was designed to investigate this apparent association between rectal lactate concentrations and mortality. However, the observations could not be reproduced in patients with septic shock for less than 24 h and the concentrations of lactate in the rectal lumen and arterial lactate were both lower than in the previous study [13]. One possible explanation for this discrepancy could be that in the first study [13] most patients had their rectal equilibrium dialysis when they had had septic shock for 48–72 h. It could be speculated that there is a “delay” in time in the development or change in the concentrations of rectal lactate. Interestingly, Poeze et al. [18] did an excellent study in 28 critically ill patients where they found that regional variables (gastric mucosal pH and indocyanine green clearance) were better to predict outcome than global hemodynamic parameters, but only after initial stabilisation, typically at least 12 hours.

 However, the data of this present study cannot support such a time-dependent change or development in the concentrations of lactate in the rectal lumen in patients with septic shock.

 Were the patients in this present study less severely ill? It seems unlikely since the SAPS II and SOFA scores were high, but they did have arterial concentrations of lactate in the lower range compared to other studies [13, 19–21]. However, such lower concentrations of arterial lactate have been observed by others in both nonsurviving and surviving patients with sepsis [18]. Also, we did not see the difference in the arterial lactate concentrations between nonsurvivors and survivors, which have been observed in other studies [18–21], but our study was not specifically designed to investigate arterial lactate concentrations. An important difference could be that the values of arterial lactate reported in our data is not strictly admission values or 24 h values but the corresponding arterial lactate concentrations at the time of rectal equilibrium dialysis which was performed at any time from 6–24 h after onset of shock.

 Because we did not observe any change in the rectal concentrations of lactate in neither the group of nonsurvivors or survivors we analysed if there were any difference in disease severity between the individual groups of patients with increasing or decreasing/no change concentrations of luminal lactate in the rectum. No such difference were found regarding SAPS II score, SOFA score at inclusion or day 5, but the actual increases or decreases in rectal lactate concentrations were small.

 Perhaps most importantly, we did not observe any difference in the concentrations of lactate in the rectal lumen compared with the arterial concentrations, a finding which also contrasts our earlier observations in septic patients [12, 13, 16]. If the concentrations of lactate in the rectal lumen are no different than arterial concentrations of lactate, no more information is gained by using this method trying to assess metabolic dysfunction of the gut.

 Are luminal concentrations of lactate a valid marker of metabolic dysfunction? Animal studies indicates this as concentrations of lactate in the intestinal lumen has been studied in animal models of occlusive gut ischaemia [22–25] and was found to be a more sensitive marker of hypoperfusion-induced intestinal metabolic dysfunction compared to lactate levels in blood or intestinal serosa or mucosa.

 There are many different techniques available for assessing blood flow and the metabolic state of the gut [5–8]. We believe our method of assessing luminal lactate in the rectal lumen is valid based on our previous studies in septic shock [12, 13, 16, 17], cardiac surgery [26], and the earlier studies by others on inflammatory bowel disease [9–11]. Thus, Perner and coworkers [26] found a 2- to 3-fold increase in concentrations of lactate in the rectal lumen in patients during coronary artery bypass grafting (CABG) with cardiopulmonary bypass as compared to off-pump CABG and healthy subjects. Similar results have been obtained by others. Using a microdialysis catheter Solligård and coworkers found a 10-fold increase in luminal rectal lactate in patients during cardiopulmonary bypass [27]. In our opinion these observations support the notion that luminal concentrations of lactate in the rectum could be a marker of ischaemia or metabolic dysfunction.

 So, could our results reflect that the patients in both our study in early septic shock [14] and the present study did not have hypoperfusion or metabolic dysfunction of the gut and that the patients in our earlier studies did have detectable metabolic dysfunction or hypoperfusion? Unfortunately neither of the studies were designed to evaluate this issue further, since we opted for a more simple study design in order to better facilitate enrolment of patients. But this should be an important question to address in future studies.

## 5. Conclusion

 Our study showed no change or development in the concentrations of lactate in the rectal lumen over the first 3 days in patients with septic shock or any difference between nonsurvivors and survivors. We found no difference between luminal rectal and arterial concentrations of lactate at any point. At present, the role of rectal equilibrium dialysis outside experimental trials is not defined and needs further investigation, ideally in studies also evaluating other methods of assessing gut metabolic function or blood flow.

##  Conflict of Interests 

The authors declare that they have no conflict of interests.

## Figures and Tables

**Table 1:** Characteristics of 22 septic shock patients. Medians (25th–75th percentiles) or numbers (percentage).

	Nonsurvivors	Survivors	*P*
	(*n* = 5)	(*n* = 17)	
Age (years)	68 (51–75)	65 (49–68)	0.43^a^
Male/female	3/2	12/5	1.00^b^
Focus of infection			
Pulmonary	1 (20%)	7 (41%)	0.61^b^
Abdominal	1 (20%)	3 (18%)	1.00^b^
Other or unknown	3 (60%)	3 (18%)	0.10^b^
Infectious agent			
Gram-negative	1 (20%)	2 (12%)	1.00^b^
Gram-positive	3 (60%)	10 (59%)	1.00^b^
Both	0 (0%)	1 (7%)	1.00^b^
Fungi, virus or unknown	1 (20%)	3 (18%)	1.00^b^
SAPS II	64 (56–75)	48 (39–70)	0.15^a^
SOFA score at inclusion	12 (9–16)	12 (9–14)	0.78^a^
Shock duration at	11 (8–17)	12 (9–18)	0.61^a^
inclusion (hours)			

^a^Mann-Whitney test

^b^Fisher's exact test.

**Table 2:** Daily parameters in 22 patients with septic shock stratified by survival.

	Nonsurvivors			Survivors		
	(*n* = 5)			(*n* = 17)		
	Day 1	Day 2	Day 3	Day 1	Day 2	Day 3
Heart rate (bpm)	94	92	80	90	90	88
	(88–114)	(80–125)	(70–95)^a^	(82–96)	(75–107)	(81–104)
Sinus rhythm	5/5	4/5	3/3	14/17	12/17	10/12
Atrial fibrillation	0/5	1/5	0/3	3/17	5/17	2/12
MAP (mmHg)	76	85	75	74	79	82
	(67–80)	(78–94)	(73–108)^a^	(70–79)	(75–85)	(71–92)
Noradrenaline dose	0.34	0.04	0.00	0.12	0.06	0.00
(*μ*g/kg/min)	(0.12–0.49)	(0.01–0.09)	(0.00–0.03)^a^	(0.09–0.16)	(0.00–0.22)	(0.00–0.15)
ScvO_2_ (%)	79	78	73	74	76	76
	(73–84)	(75–80)	(62–76)^a^	(68–80)	(70–80)	(72–80)
IAP (mmHg)	14	16^#^	12	12	13	12
	(11–16)	(15–17)	(11–14)^a^	(11–15)	(9–15)	(8–17)
Lactate, arterial	1.9	1.5	1.4	1.8	2.0	1.4
(mmol/L)	(1.6–8.2)	(1.2–3.7)	(1.4–3.4)^a^	(1.4–3.4)	(1.3–2.6)	(1.1–2.2)
Lactate, rectal lumen	2.4	3.2	2.9	2.1	2.1	2.3
(mmol/ L)	(1.3–7.5)	(1.7–4.2)	(1.5–3.0)^a^	(1.2–4.4)	(1.1–3.4)	(0.9–3.0)
Delta-lactate	−0.25	0.60	0.20	0.45	−0.10	0.30
(rectal-arterial (mmol/ L))	(−1.0–0.3)	(−0.4–2.0)	(−0.5–1.7)	(−0.5–1.2)	(−1.1–2.0)	(−0.4–1.3)

Values are medians (25th–75th percentiles). ^#^ *P* = 0.02 compared with IAP day 2 in survivors. No other significant differenceswere found using the Mann Whitney test (comparing values between groups on specific days) or Wilcoxon's signed rank test or Friedmans's test (comparing paired values within groups over 2 or 3 days, resp.). ^a^Range  since *n* = 3.

**Table 3:** SAPS II score, SOFA score at inclusion and day 5 of patients with an increase or decrease/no change In luminal rectal lactate from day 1 to day 2 or from day 2 to day 3, respectively.

	From day 1 to day 2		*P*	From day 2 to day 3		*P*
	Increase in	Decrease in		Increase in	Decrease in	
	rectal lactate	rectal lactate		rectal lactate	rectal lactate	
	(*n* = 11)	(*n* = 11)		(*n* = 7)	(*n* = 8)	
SAPS II	64	46	0.07	53	55	0.96
	(48–74)	(28–53)		(46–74)	(43–72)	
SOFA	12	10	0.14	13	13	0.78
(at inclusion)	(11–15)	(8–13)		(10–14)	(10–16)	
SOFA	8	8	0.67	8	7	0.54
(day 5)	(4–13)	(6–11)		(8–13)	(4–13)	

Values are medians (25th–75th percentiles). Statistical analysis comparing values between patients with an increase or a decrease/no change in rectal luminal lactate were done using the Mann-Whitney test.
